# A Magnetron Plasma Arc Fusion Identification Study Based on GPCC-CNN-SVM Multi-Source Signal Fusion

**DOI:** 10.3390/s25102996

**Published:** 2025-05-09

**Authors:** Yeming Zou, Dongqian Wang, Yuanyuan Qu, Hao Liu, Aiting Jia, Bo Hong

**Affiliations:** 1 School of Mechanical Engineering and Mechanics, Xiangtan University, Xiangtan 411105, China; yemingzou@163.com (Y.Z.); dqianwang200124@163.com (D.W.); 13016173718@163.com (H.L.); 2 School of Mechanical Engineering, Hunan University of Science and Technology, Xiangtan 411100, China; qyyworld@hnust.edu.cn; 3 Engineering Research Center of Complex Track Processing Technology & Equipment, Ministry of Education, Xiangtan University, Xiangtan 411105, China

**Keywords:** multi-pole magnetron, plasma welding, arc signal, arc pressure signal, melt penetration identification

## Abstract

Plasma arc welding (PAW) is commonly employed for welding medium and thick plates due to its capability of single-side welding and double-side forming. Ensuring welding quality necessitates real-time precise identification of the melting state. However, the intricate interaction between the plasma arc and the molten pool, along with substantial signal noise, poses a significant technical hurdle for achieving accurate real-time melting state identification. This study introduces a magnetically controlled method for identifying plasma arc melt-through, which integrates arc voltage and arc pool pressure. The application of an alternating transverse magnetic field induces regular oscillations in the melt pool by the plasma arc. The frequency characteristics of the arc voltage and pressure signals during these oscillations exhibit distinct mapping relationships with various fusion states. A hybrid feature extraction model combining gray correlation analysis (GRA) and the Pearson correlation coefficient (PCC) is devised to disentangle the nonlinear, non-smooth, and high-dimensional repetitive features of the signals. This model extracts features highly correlated with the fusion state to construct a feature vector. Subsequently, this vector serves as input for the fusion classification model, CNN-SVM, facilitating fusion state identification. The experimental results of melt-through under various welding speeds demonstrate the robustness of the proposed method for identifying melt-through through magnetic field-assisted melt pool oscillation, achieving an accuracy of 96%. This method holds promise for integration into the closed-loop quality control system of plasma arc welding, enabling real-time monitoring and control of melt pool quality.

## 1. Introduction

Welding technology is a crucial process in modern industrial manufacturing with diverse applications in aerospace [1], shipbuilding [2], medical equipment [3], and power equipment [4]. The technical advancement of welding serves as a significant metric for assessing national industrial development [5]. Due to its high energy density, substantial fusion depth, minimal heat-affected zone, rapid welding speed, and other notable benefits [6], plasma arc welding is particularly indispensable for medium-thickness plate welding and high-precision welding [7,8,9]. The fusion depth, a pivotal parameter reflecting weld quality, directly influences the mechanical properties and longevity of the product. Therefore, real-time monitoring and precise prediction of fusion depth hold substantial technical value for enhancing welding quality and optimizing process parameters.

Research in dynamic monitoring of welding processes primarily focuses on three detection methods: arc sound signal, visual information, and melt pool oscillation characteristics. Arc sound detection research follows a comprehensive technical pathway from signal pre-processing and feature extraction to intelligent recognition, offering a distinct advantage in time domain resolution. Cui et al. [10] utilized the spectral subtraction method (SNS) to suppress noise and extract pure arc acoustic signals effectively. Di Wu et al. [11,12] developed a dimensionality reduction fusion method based on t-stochastic neighbor embedding (t-SNE), a deep belief network (DBN), a hybrid framework of a convolutional neural network (CNN), and an extreme learning machine (ELM) to realize collaborative analysis of visual–acoustic signals. Gao et al. [13,14,15,16] innovatively applied time–frequency analysis by Short-Time Fourier Transform and a convolutional neural network (STFT-CNN), subjective evaluation modeling by Multiple Dimensional Scaling (MDS), and auditory simulation to process the arc sound signals to significantly improve the recognition accuracy. Furthermore, Zhang et al. [17] and Luo et al. [18] processed acoustic signals by optimizing deep learning methods using optimized CNNs and Convolutional Long Short-Term Memory (CNN-LSTM) networks, respectively. The time–domain resolution characteristics of arc sound signals have strong environmental adaptability and are suitable for welding quality monitoring. However, the inherent defect of environmental noise interference limits the effectiveness of this technique for industrial applications, motivating researchers to move toward multi-sensor fusion to improve system robustness.

Visual sensing provides multi-dimensional feature information necessary for melt depth monitoring through the capture of melt pool morphology and temperature field distribution. Building upon the foundations of traditional image processing, Chen et al. [19] developed a prediction model that utilizes the variation of reflected light projection from the melt pool. Li et al. [20] found that the recognition accuracy of geometric features was 93%, and Liang et al. [21] utilized the support vector machine (SVM) regression analysis to examine the surface topographic parameters of the melt pool. The feature extraction capability was enhanced by deep learning. Baek et al. [22,23] successively developed CNN feature extraction and Residual Network (ResNet) semantic segmentation models to extract accurate melt pool shapes from weld pool images. Jia et al. [24] proposed a deep learning-based prediction model for plasma arc welding (PAW) melt depth and locked hole state. Yu et al. [25] and Zeng et al. [26] innovatively combined a lightweight convolutional neural network (ShuffleNetV2) with an online sequential extreme learning algorithm with a forgetting mechanism (FOS-ELM). With the fusion of multi-dimensional data features, Li et al. [27] correlated the plasma plume and spectral properties with the melt depth state. Zhang et al. [28] combined the locked aperture area (KA) and time–frequency domain properties of the plasma plume, and Zeng et al. [29] integrated optoelectronic signals with visual information, thereby obtaining multi-dimensional data fusion of morphology and temperature. YuMing Zhang et al. [30] developed a passive vision sensing system with two cameras to monitor the top and bottom information of the molten pool simultaneously and achieved a weld melt penetration accuracy of 96.35% through deep learning. YuMing Zhang et al. [31] also reviewed the deep learning based in situ monitoring study of weld melt penetration, including welding images and arc acoustic signals. Visual sensing can provide multi-dimensional information such as melt pool geometry, temperature field distribution, reflected light characteristics, etc. However, under AC magnetron conditions, the arc light is prone to fluctuations and strong reflective workpiece interference, and the vibration caused by magnetism will interfere with the electronic components of the visual sensor, affecting the image quality, so it has become a trend to combine the information provided by a variety of sensors to reduce the limitations of a single sensor and improve melt penetration monitoring. Therefore, combining information from multiple sensors has become a trend to reduce the limitations of a single sensor and improve the accuracy and reliability of melt penetration monitoring. 

The high dynamic range visual sensor mentioned in the above study, with an illumination intensity of 20 Mw and a signal-to-noise ratio of more than 24 dB, and equipped with a special light source and filter can capture high-quality images, but its applicability is limited because it still affects the clarity of the image and the accuracy of feature extraction when there are strong reflections or background noise. The arc signal is predominantly determined by the variation characteristics of the arc current and voltage during the welding procedure. It establishes a mapping relationship between the melt pool oscillation and the melt penetration state. Additionally, the arc signal manifests distinct time domain and frequency domain characteristics. This study explores the identification of characteristic frequency bands in the context of arc signal analysis. Cui et al. [32] found a significant correlation between the 2–4 kHz frequency band and the state of welding melt depth. Xu Sai et al. [33] analyzed the relationship between the vibration period of small holes and the depth of small holes. According to the threshold determination method, Zhang et al. [34] proposed the melting depth monitoring based on the arc voltage fluctuation amplitude (ΔUk) to establish the basis for judging the full penetration with ΔUk > 1.5 V, and Bai et al. [35] proposed a critical penetration threshold of ΔU = 4.10 V to monitor the melting penetration of pulsed gas metal arc welding (GMAW-P). In the domain of intelligent identification algorithms. Wang et al. [36] fused image features and combined welding current and voltage data, extracting temporal sequences and spatial features using improved convolutional blocks and attention mechanisms. She et al. [37] used a dimensionality reduction method to extract spectral features based on single-sensor and multi-sensor melt penetration prediction models, respectively. Anh Van Nguyen et al. [38] monitored the keyhole formation and detected the fusion state by observing eddy currents and fluid flow in the melt pool. Zou et al. [39] proposed a melt depth prediction method based on the relative fluctuation factor (CRF), which was used as feedback for model prediction control to realize the melt depth control.

The application of an alternating magnetic field has been demonstrated to regulate the melt pool flow, redistribute the heat [40], enhance the metal heat exchange, and reduce the temperature gradient of the melt pool [41] to optimize the joint depth of fusion. Jun Zhou et al. [42] designed an alternating magnetic field generator to assist laser welding of aluminum alloys to improve the penetration of the weld gap bridging. Fuyun Liu et al. [43] found that the varying flux densities within the alternating magnetic field influenced the laser–MIG composite welding of 316 stainless steel joints, particularly affecting the weld depth. Liming Liu et al. [44] employed a parallel alternating magnetic field to facilitate the double tungsten silver electrode indirect arc welding process, resulting in a substantial enhancement in weld depth. The alternating magnetic field has been demonstrated to serve as an effective method for altering the arc energy distribution. This, in turn, serves the purpose of regulating the heat input and pressure distribution. The arc pressure contains information related to the arc energy. It can reflect the dynamic behavior and penetration state of the arc and the molten pool. Compared to acoustic signals, visual information, and arc signals, arc pressure is less affected by external factors, such as electromagnetic interference and arc light interference, in the welding process. It can more stably reflect the intrinsic state of the welding process in a complex welding environment.

Solving the plasma arc welding process involves nonlinear and multi-physical field coupling; strong arc light, metal spatter, and other factors will interfere with the signal acquisition, resulting in difficulties in real-time monitoring of the fusion state in the plasma welding process, as well as compensating for the lack of simple information dimension of the arc signal. This paper proposes an arc voltage and arc pressure multi-source information fusion depth of fusion monitoring method based on multi-pole magnetron technology. Specifically, it establishes a multi-information sensing system of a magnetron sensor, pressure sensor, and arc sensor, actively induces the periodic oscillation of the arc and enhances the oscillation effect of the melt pool, synchronously collects the arc electric signal and arc pressure signal, and constructs an intelligent identification model for multi-source information fusion. The main innovations are as follows:

(1) It is proposed that more significant melt depth feature signals be induced by the periodic oscillation of the melt pool through the multipolar alternating magnetic field.

(2) A multi-source signal melt-through identification method based on the bimodal signal fusion complementary to the arc voltage signal and the arc pool pressure signal is proposed.

(3) The advantages of the GRA-PCC are to be combined in the small-sample, nonlinear, and high-dimensional pattern classification models, and an adapted CNN-SVM melt penetration identification model is to be constructed.

In comparison with the conventional single-signal monitoring technique, this methodology can enhance the precision and resilience of melt penetration state identification. It offers a novel approach to implementing intelligent closed-loop control of welding quality.

## 2. Magnetron Plasma Arc Welding Multi-Information Sensor System

### 2.1. Multi-Source Signal Fusion Experimental Apparatus

**Figure 1:**
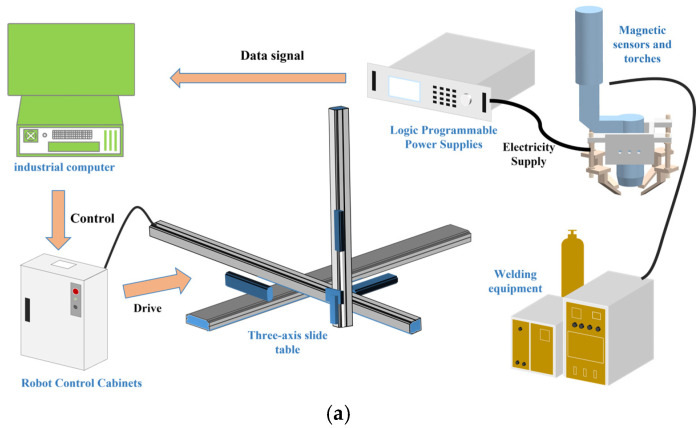

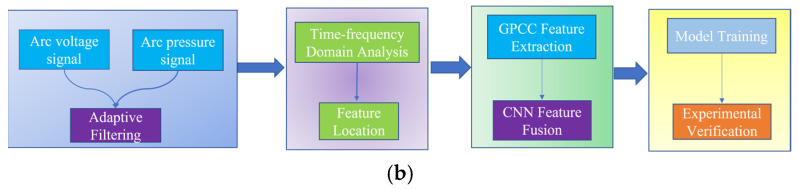
(**a**) Multi-source signal fusion penetration test platform; (**b**) workflow graph.

**Figure 2:**
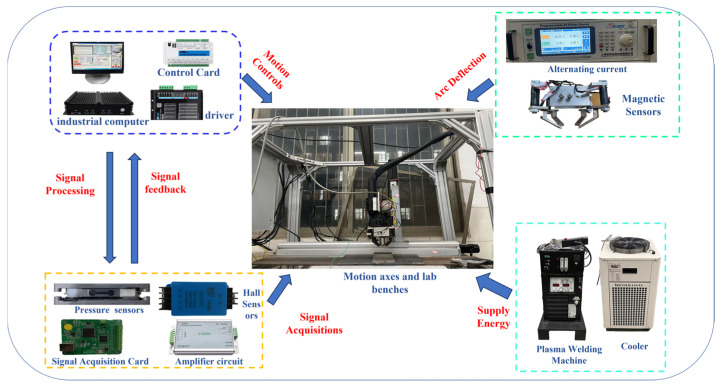
Magnetron plasma arc welding multi-information sensor system integration.

As illustrated in Figure 1, the multi-source signal fusion test platform consists of four primary modules: a multi-pole magnetic control arc system, a robot motion control system, a plasma welding machine, and a signal acquisition system. The multi-pole magnetic control arc system generates an alternating magnetic field to control the arc oscillation and collect the corresponding arc signals. This system consists of a variable-frequency power source, known as the HY-LCS2P, manufactured by Hangyu Company (Beijing, China), and four symmetrical magnetic poles. The welding power was provided by a Tetrix 452 Synergic precision plasma welder from EWM, Dortmund, Germany. Hall sensors (CHV-50VD, Sensor Electronics Company, Beijing, China) and pressure sensors (cantilever type, VH130,Viste, Shenzhen, China) were then utilized to collect arc voltage and pressure signals. The motion control system is comprised of a control cabinet and a three-axis sliding table (model JD45C, manufactured by the Shanghai Aluminum Company of Shanghai, China). The arc morphology and molten pool observations were performed using a high-speed camera (model Aguteye, manufactured by Ketianjian, Changsha, China) that samples at 500 frames per second with a resolution of 640 × 512. The system integration is illustrated in Figure 2.

### 2.2. Experimental Parameters for Multi-Source Signal Fusion Identification

The results of the magnetron plasma arc penetration experiment show that the arc swing and molten pool oscillation increase with the increase in the excitation current and excitation frequency, and the oscillation characteristics of the signal become more obvious. The welding parameters (including welding current, welding height, welding speed, and gas flow rate) were all determined through a large number of process tests and orthogonal tests for optimization. The specific values are listed in Table 1. A tungsten needle with a diameter of 4.0 mm was used in the experiment. The workpiece material was 304 stainless steel with dimensions of 200 mm × 100 mm × 5 mm. After determining the welding process, when the excitation current varies in the range of 0–3 A and the excitation frequency is adjusted in the range of 2–6 Hz, the arc stability shows a significant correlation with the signal characteristics. Based on the systematic analysis of the test data, it was finally determined that, on the premise of ensuring the stability of the arc, the excitation current of 3 A and the excitation frequency of 6 Hz were selected as the optimal magnetic field parameters to obtain the most obvious oscillation signal characteristics.

### 2.3. Multipolar Magnetron Arc Oscillation Model and Behavioral Characterization

Multi-pole magnetron arc swing simulation parameters and the actual welding parameters consistent with the simulation of the basic assumptions are as follows:

(1) The plasma is a fully ionized conductive fluid, and its conductivity is related to the temperature and gas composition, and in the local thermal equilibrium state, the electron temperature is equal to the temperature of the ions.

(2) The Lorentz force of the magnetic field on the plasma is significant, while the inertial and viscous effects of the plasma are relatively small and can be ignored.

(3) Plasma heat transfer depends mainly on electrons, whose thermal conductivity is proportional to the temperature cube.

(4) The magnetic field is generated by a multi-pole magnetron device with a known external magnetic field distribution.

**Figure 3:**
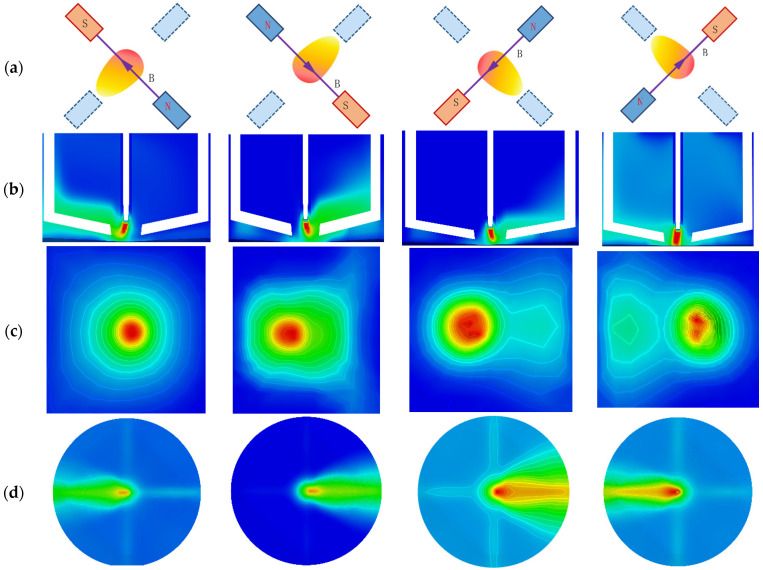

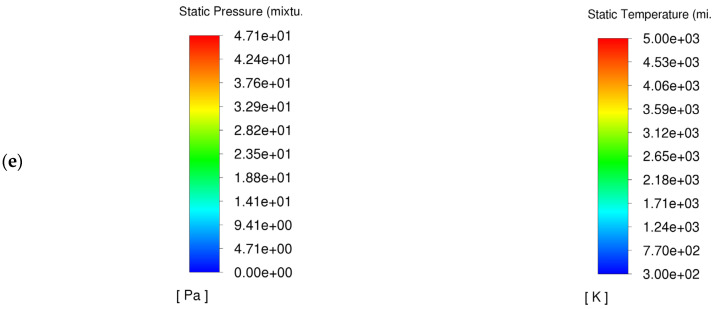
Multi-pole magnetically controlled arc swing where the arc is controlled: (**a**) magnetic field sequence, (**b**) arc swing, (**c**) pressure distribution, (**d**) temperature distribution, (**e**) Pressure and temperature distribution scale.

As demonstrated in Figure 3a, the excitation power supply is applied to the copper coil AC, thereby generating an AC magnetic field. In the context of multiple magnetron welding, the external magnetic field exerts a driving force on the periodic oscillation of the arc on the surface of the molten pool through the Lorentz force (see Figure 3b). This oscillation significantly alters the heat input and hydrodynamic properties of the molten pool, affecting the pressure distribution (Figure 3c) and temperature distribution (Figure 3d) at the surface of the molten pool. The oscillating arc leads to a more uniform distribution of heat on the surface of the molten pool, a reduction in the temperature gradient within the molten pool, and concurrently drives the liquid metal within the molten pool to form a complex vortex structure. This enhances momentum transfer, reduces local pressure concentration, and facilitates the formation of a stable melt-through shape by the molten pool. The arc length change and pressure fluctuation caused by the arc swing can be obtained through the sensor arc pressure and pressure signals. Analysis of the characteristics of these signals can determine the penetration state and analyze the melt depth.

### 2.4. SVM Model Structure and Principle

The support vector machine (SVM) is a widely utilized machine learning algorithm for text categorization, data classification, computer science, and other practical applications in the field [45]. The SVM algorithm is based on the process of nonlinear problems by feature mapping and kernel functions, and it is classified by finding the best hyperplane to maximize the interval. The fundamental objective of the SVM is to identify a hyperplane that optimizes the separation between data points from distinct categories while maximizing the interval between those data points that extend closest to the decision boundary, also known as the support vector. This hyperplane finds application in the classification of new data points into distinct categories.

The classification decision function of the SVM can be represented as follows:

(1) f(x)=sign(w·x+b)

where *x* is the feature vector of the input sample, *W* is the normal vector of the hyperplane, *b* is the bias term, *sign* is the sign function that determines the class to which the data point belongs according to the sign of the function, and *w·x + b* is the separating hyperplane.

(1) Linear kernel function. The linear kernel function is mainly used in the case of linear differentiation and has the advantage of fewer arguments and faster speed. The representation is as follows:

(2) $$K(x_{i},x_{j})=x_{i}^{T}*x_{j}$$

where *x_i_* and *x_j_* denote the feature vectors of the data for which classification is attempted—the transpose vector—and *K*(*x_i_*_,_ *x_j_*) denotes the inner product between the two feature vectors obtained by linear kernel computation.

(2) Polynomial kernel function. The polynomial kernel function can map the low-dimensional input space to the high-dimensional feature space. It is suitable for orthogonal normalized data, which affects the value of the kernel function, and solves nonlinear problems. It is represented as follows:

(3) $$K(x_{i},x_{j})={\left(x_{i}^{T}*x_{j}+c\right)}^{d}$$

where *C* is a constant and *d* is the order of the polynomial kernel.

(3) Radial Basis Kernel Function (RBF). The RBF is one of the most commonly used kernel functions in the SVM, usually for nonlinear data. It is a local kernel function that maps the samples to a higher-dimensional space, has fewer parameters than polynomial kernel functions, and has better immunity to noise in the data, but it is sometimes slow to compute. It is expressed as

(4) $$K(x_{i},x_{j})=\exp(\frac{-||x_{i}-x_{j}|{|}^{2}}{2\delta^{2}})$$

where || || denotes the norm of the vector (usually the Euclidean distance) and *σ* is a parameter of the RBF that controls the similarity between sample points.

## 3. Multi-Source Signal and Fusion State Analysis Method

### 3.1. Multi-Source Signal Fusion State Mapping

There are distinctive melt pool oscillation modes corresponding to varying fusion states, and, therefore, the fusion states of the melt pool can be extracted by employing signal processing.

**Figure 4:**
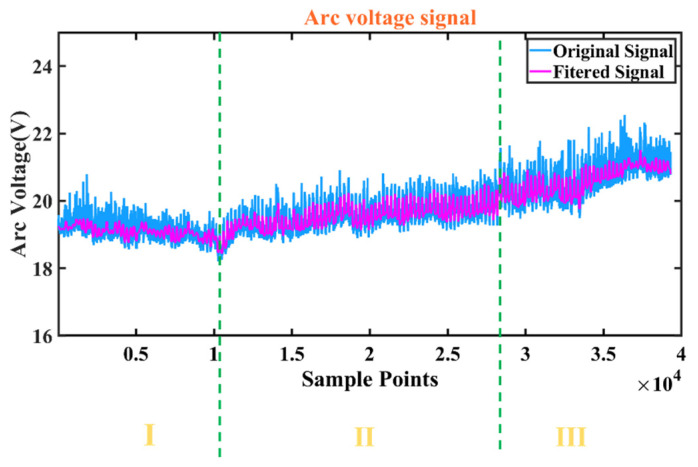
Arcing voltage signal.

**Figure 5:**
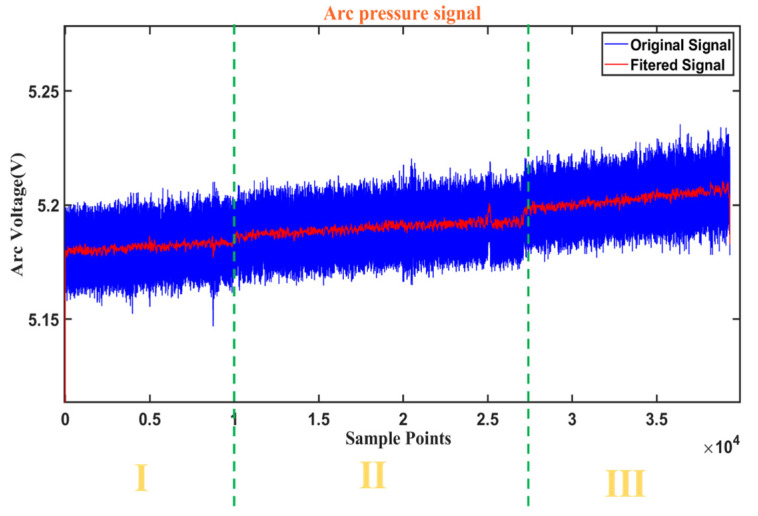
Arc pressure signal.

**Figure 6:**
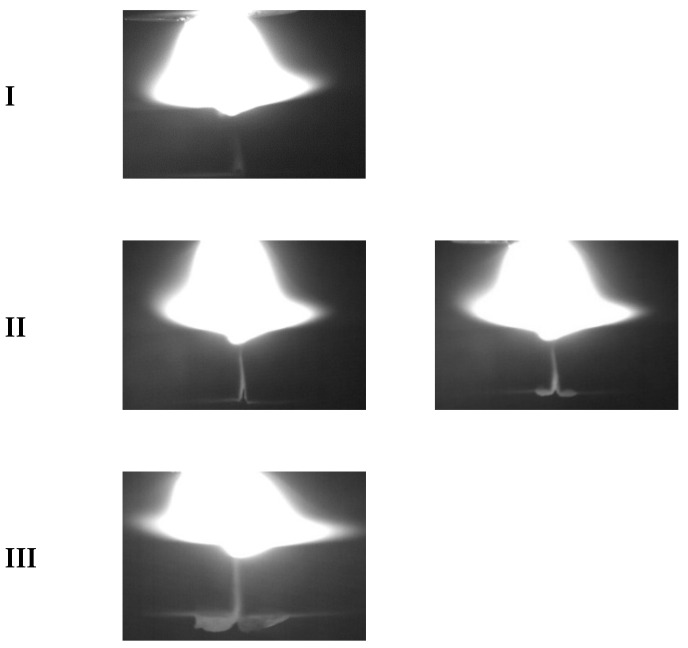
Changes in the melt-through.

Figure 4 and Figure 5 show the variation with time of the synchronized arc and pressure signals obtained from Hall sensors and cantilever beam pressure sensors, respectively, with a sampling frequency of 1000 Hz and 1000 samples for 1 s. Figure 6 shows the fusion state at three different stages. At stage I, the melt penetration is shallower and the melt pool is narrower. At stage II, the melt penetration is deeper and the melt pool is wider. At stage III, the melt penetration continues to deepen and the melt pool continues to widen.

The mean values of the three stages of the arc signal in Figure 4 are 19.12 V, 19.52 V, and 21.06 V, and the variances are 0.06, 0.17, and 0.23, respectively, indicating that the fluctuations of the arc signal are gradually increasing, and the higher variance values indicate that the fluctuations of the arc energy are more significant. The mean values of the three stages of the arc pressure signal in Figure 5 are 5.18 V, 5.19 V, and 5.20 V, respectively, the variance of the three stages varies around 8 × 10^−6^ with small fluctuations, and the force and dynamics of the melt pool remain relatively the same at different stages. At different sampling point intervals (I, II, III), the arc voltage reflects the stability and energy input of the arc at different stages. The arc pressure reflects the force and dynamic change in the melt pool at different stages of the arc. In stage I, the arc voltage and pressure fluctuate less, indicating a more stable arc with less energy input and force. This corresponds to the condition shown in Figure 6, where the melt penetration is shallower and the melt pool is narrower. In stage II, the fluctuations in arc voltage and arc pressure begin to increase, indicating that the energy input and force of the arc are increasing. This corresponds to the state of deeper melt penetration and wider melt pool in Figure 6. In stage III, the fluctuations of arc voltage and arc pressure continue to increase, indicating that the energy input and force of the arc are at their maximum. This corresponds to the state in Figure 6, where the melt penetration continues to deepen and the melt pool continues to widen.

The variations in the arc voltage and arc pressure signals are indicative of the stability of the arc, the energy input, and the role of force. These factors directly affect the state of weld penetration. Through the analysis of these signals, the depth of penetration can be predicted and controlled.

### 3.2. Time–Frequency Domain Analysis of Multi-Source Signals

**Figure 7:**
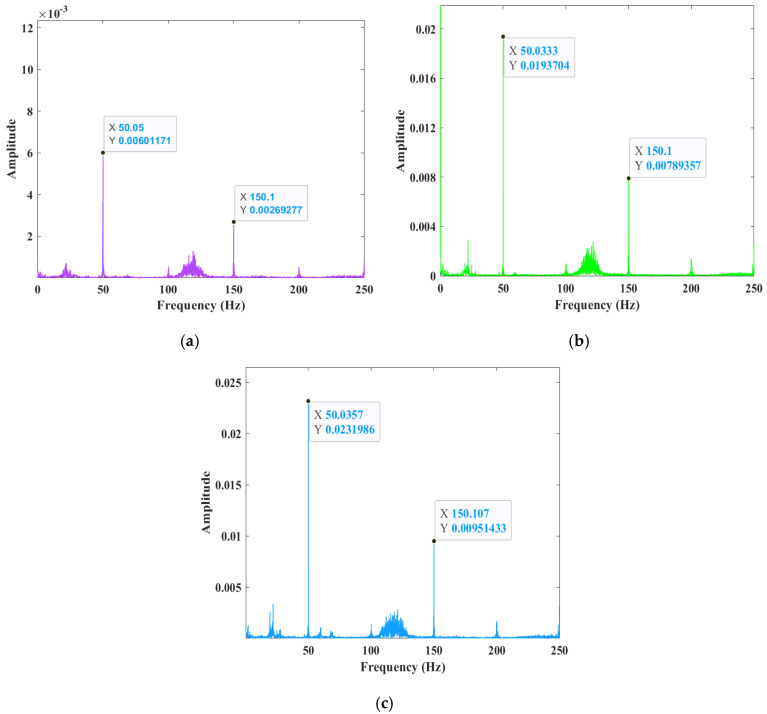
Pressure signal spectra: (**a**) unmelted; (**b**) completely melted; (**c**) overmelted.

As illustrated in Figure 7, the spectra of pressure signals I to III demonstrate notable frequency peaks at approximately 50 Hz and 150 Hz. These peaks can be associated with arc oscillation and harmonics during the welding process. The 50 Hz peaks are found to be associated with the oscillation frequency of the arc, and the higher amplitude indicates that the arc oscillates more vigorously during the welding process, resulting in deeper penetration. Peaks at 150 Hz are associated with the harmonics of the arc oscillation, reflecting more complex pool oscillations. However, the higher amplitude of the high-frequency oscillation indicates greater penetration as the pool transitions from a fully molten state to an over-molten one. The 150 Hz peaks are also found to be associated with the harmonics of the arc oscillation, reflecting more complex pool oscillations, and the higher amplitude of the high-frequency oscillation indicates greater penetration. As the pool transitions from a fully penetrated state to an over-penetrated state, the pool oscillations continue to increase, but the arc pressure fluctuations tend to stabilize. The fluctuation of the arc pressure in the time domain manifests itself as a peak at a specific frequency in the frequency domain, which can be used as a feature to identify melt-through.

**Figure 8:**
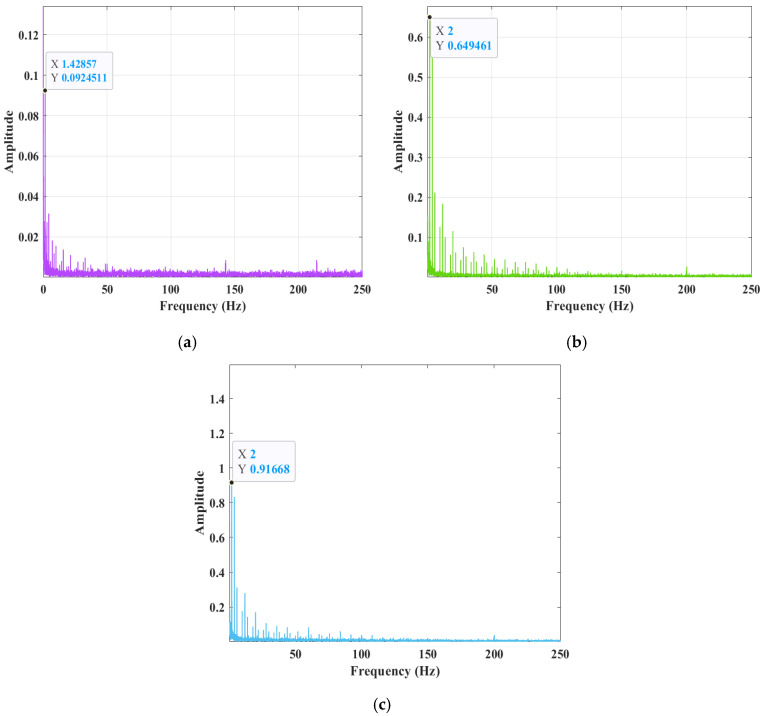
Arc signal spectra: (**a**) unmelted; (**b**) completely molten; (**c**) overmelted.

As illustrated in Figure 8, the spectrogram of the arc signal reveals the presence of harmonics, which are indicative of signal distortion. As illustrated in Figure 8a, a prominent peak emerges at 1.43 Hz, with an amplitude of approximately 0.09245, indicating a substantial energy concentration within the low-frequency range. In contrast, Figure 8b exhibits a peak at 2 Hz, with an amplitude of around 0.649461, suggesting an augmentation in the high-frequency component. Finally, Figure 8c demonstrates a peak at 2 Hz, with an amplitude of about 0.91668, indicating a sustained enhancement in the high-frequency component. The low-frequency peak is associated with the arc oscillation frequency, and its magnitude increases as the melt pool transitions from the unmelted to the overmelted state. In instances where the melt pool has not yet undergone complete melting, the molten metal is supported by the bottom of the workpiece. The arc extrusion process causes the metal’s surface to vibrate at a distinct vibrational frequency. The presence of different melting states corresponds to unique vibrational modes that can be extracted from the melting state through signal processing.

Following the characteristics of the arc signal and pressure signal during the oscillation of the molten bath under multi-pole magnetic control, 11 kinds of time–frequency domain indices are selected for the arc signal: mean value, variance, root mean square, kurtosis, skewness, maximum value, minimum value, peak-to-valley value, waveform factor, margin factor, slope, and so on. Fourteen time–frequency domain indicators are selected for the pressure signal: mean value, variance, root mean square, kurtosis, skewness, slope, centroid frequency, flatness, craziness, entropy, mean square frequency, variance frequency, 50 Hz amplitude value, 150 Hz amplitude value, and the construction of feature vectors as shown in the Table 2, wherein 50 Hz and 150 Hz amplitude values are obtained from the spectrogram of the pressure signal, where *x_i_* is the value of the detected signal and m is the number of detected sample points. In this equation, *x_i_* denotes the detected signal value and m signifies the number of detected sample points.

### 3.3. Multi-Source Signal Feature Downscaling and Fusion Under Different Fusion States

In the study of multi-pole magnetron plasma welding penetration state identification, the arc voltage signal and the melt pool pressure signal are identified as the key monitoring quantities. However, it is noted that both of these are weak target signals in a strong noise background, with nonlinear, non-smooth, and high-dimensional characteristics. This has the effect of making high-precision penetration state identification difficult. The arc voltage signal is a non-periodic random pulse train, and its transient amplitude, mean value, and power spectral density demonstrate a nonlinear relationship with the fusion state change. There are multivariate, broadband, and long correlation characteristics, accompanied by rich harmonics and strong fundamental waves, forming a complex time-varying signal-to-noise ratio environment. Concurrently, the arc pool pressure signals are synchronized but not fully consistent across different channels, which are characterized by the high degree of discrete time series. Consequently, it is imperative to extract effective feature subsets from a substantial number of time–frequency domain features and effectively fuse multi-source information to achieve high-precision melt-through state judgment.

The arc voltage signal is primarily influenced by high-frequency electromagnetic interference, while the melt pool pressure signal is affected by mechanical vibration and fluid turbulence. Consequently, the signal-to-noise ratio can be enhanced by integrating time and frequency domain features. As demonstrated in Table 3, multi-sensor fusion enhances the SNR by 7–10 dB, which is equivalent to 1/10 to 1/5 of the initial noise power value. Conversely, the single-signal SNR of the arc voltage and the melt pool pressure is constrained to 5–10 dB and 0–6 dB, respectively, which poses challenges in meeting the requirement for high-precision identification. It is imperative to extract effective feature subsets from a substantial number of time–frequency domain features and to efficaciously fuse multi-source information in order to achieve high-precision judgment of the melting state.

#### 3.3.1. Gray Relational Analysis (GRA)

Gray relational analysis (GRA) is a methodological approach for the analysis of the degree of correlation among factors. It is most frequently employed for correlation analysis and decision support among multiple factors. It is founded on the theory of gray systems and quantifies the degree of similarity and correlation between factors through quantitative analysis. GRA has found extensive application in a variety of fields, including analytical forecasting [46], feature selection [47], and system optimization [48], to name but a few. It plays a pivotal role in practical applications. It assists researchers and decision-makers in comprehending the interrelationships among multiple factors, evaluating the impact of factors on objectives, and identifying the optimal solution or decision-making.

The gray correlation degree model is utilized to calculate the correlation between variables. The gray correlation coefficient is indicative of the similarity or proximity between the variables. The calculation of the gray system correlation coefficient, denoted by ζi, and the degree of relatedness, symbolized by hi, is achieved through the application of Equations (5) and (6), respectively, as follows:

(5) $$\xi_{i}=\frac{\min_{i,k}\Delta X_{i}(k)+\rho \max_{i,k}\Delta X_{i}(k)}{\Delta X_{i}(k)+\rho \max_{i,k}\Delta X_{i}(k)}$$

(6) $$h_{i}=\frac{1}{n}\sum_{k=1}^{n}\xi_{i}(k)$$

where min*_i,k_*ΔX*_i_*(k) and max*_i,k_*ΔX*_i_*(k) are the minimum and maximum values of the difference of all data points in all comparison and reference series, respectively. The correlation degree hi is the average value of the correlation coefficient ξi(k) over all data points, and the correlation degree values are all between 0 and 1.

**Figure 9:**
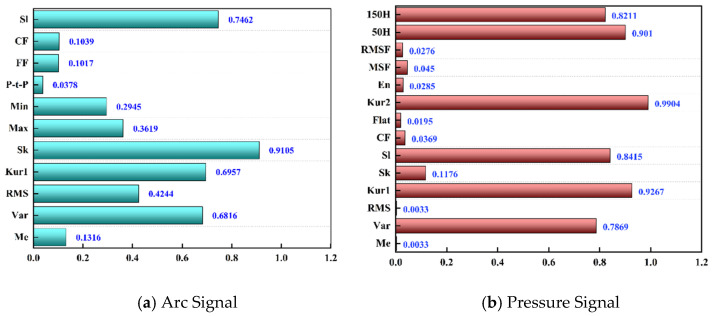
Correlation between different types of GRA features and fusion state classification results.

As illustrated in Figure 9, the results of the GRA analysis of the arc voltage signal, melt pool pressure signal, and their corresponding melt penetration classification are presented. As is evident from the figure, there are four types of features of the arc voltage signal with a high GRA correlation degree for melt penetration classification, while there are six types of features of the melt pool pressure signal with a strong GRA correlation for melt penetration classification. It is noteworthy that GRA, as a nonlinear correlation calculation method based on information theory, is well suited to characterize the complex, nonlinear, and non-deterministic correlation between various signal features and the classification results of the fusion state. Consequently, GRA can more accurately reflect the nonlinear coupling relationship between the signal features and the classification of the fusion state in the welding process.

#### 3.3.2. Pearson Correlation Analysis (PCC)

GRA is utilized in the calculation and analysis of data samples, though it should be noted that this approach can only yield basic statistical information regarding the relationship between features and the target. It fails to provide insights into the linear and nonlinear coupling effects between features. To gain a more nuanced understanding of the interactions between these features and their impact on the melting situation, GRA should be employed in conjunction with other analytical methods.

Pearson correlation analysis (PCC) is a statistical analysis method used to evaluate the linear correlation between two variables. It measures the correlation between two variables with values ranging from −1 to 1 [49]. The Pearson correlation coefficient is calculated using Equation (7) as follows:

(7) $$P_{i}=\frac{\sum_{i=1}^{n}(X_{i}-\bar{X})(Y_{i}-\bar{Y})}{\sqrt{\sum_{i=1}^{n}(Y_{i}-\bar{Y}{)}^{2}}\sqrt{\sum_{i=1}^{n}(X_{i}-\bar{X}{)}^{2}}}$$

*X_i_* and *Y_i_* denote the values of the two variables and denote the means of the two variables, respectively. If p is positive, it means that there is a positive correlation between the two variables, and if p is negative, it means that there is a negative correlation between the two variables. If *| p |* is close to 1, it means that there is a strong linear relationship between the two variables. If *| p |* is close to 0, it means that there is a weak or no linear relationship between the two variables. The comparative analysis of GRA and PCC as shown in the Table 4.

**Figure 10:**
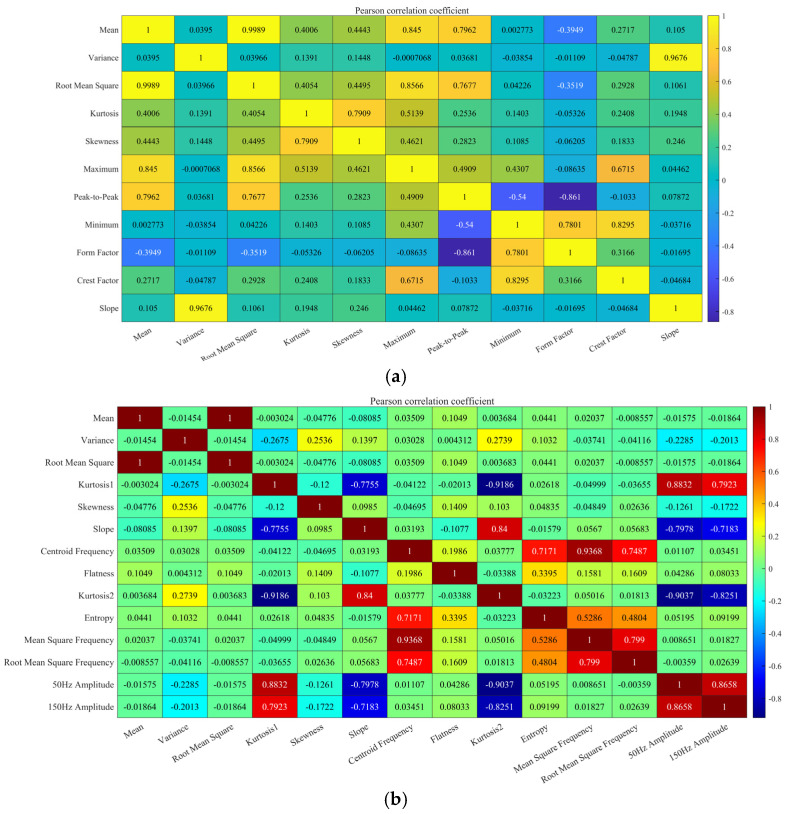
PCC thermogram distribution: (**a**) arc signal; (**b**) pressure signal.

As illustrated in Figure 10, the heat map of the feature correlation, as determined by the Pearson correlation coefficient, utilizes color shades to denote the linear correlation between the features and the fusion state. The arc signal feature correlations are concentrated in the medium range (|ρ| < 0.4~0.6), while the pressure signal feature correlations are more distinct, with strong correlations (|ρ| > 0.7) for some features.

**Figure 11:**
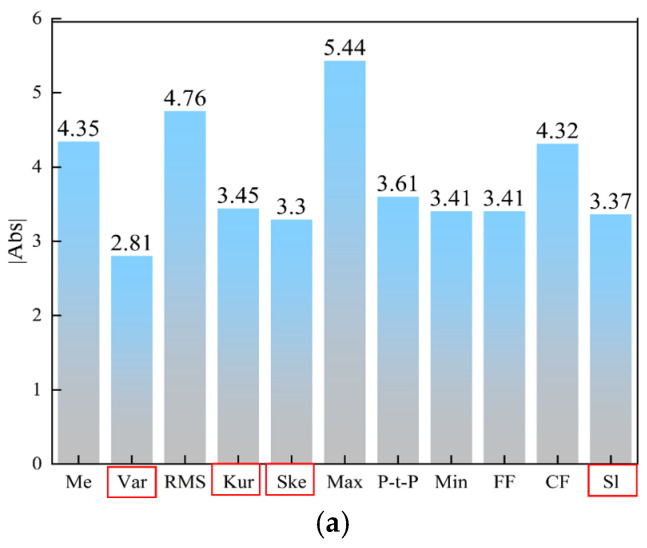

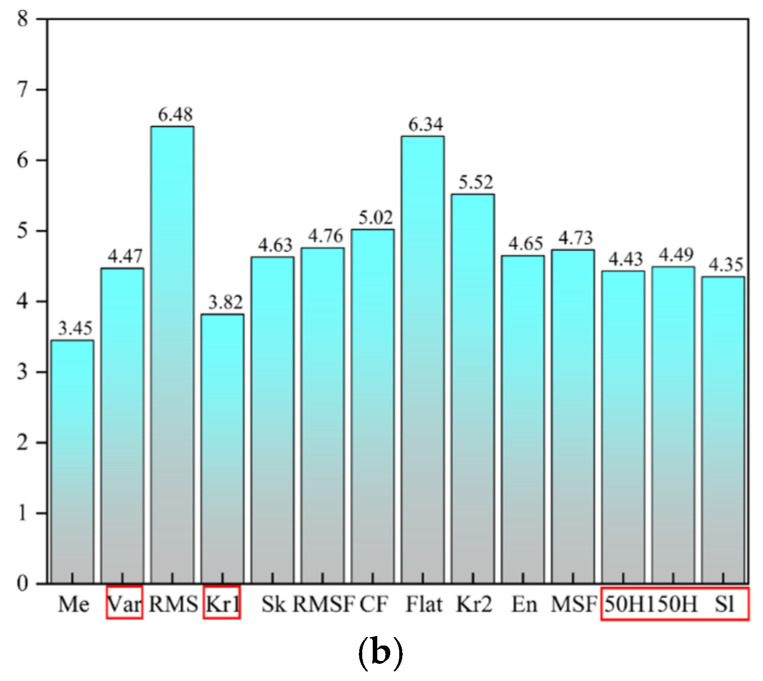
PCC feature optimization results: (**a**) arc signal; (**b**) pressure signal.

To further reflect the overall degree of correlation, Figure 11 shows the distribution of the sum of the absolute Pearson coefficients (Σ|ρ|) for each type of feature, with the red squares marking the low correlation features. Four feature types exhibit significantly low correlation (Σ|ρ| < 3.5) in the arc signal, while five additional independent feature types (Σ|ρ| < 4.5) are observed in the pressure signal. However, these latter types are disregarded due to their inherently low correlation and the information they contain about the melt pool. Consequently, it is necessary to reserve them for study at the feature combination stage.

#### 3.3.3. GPCC Multi-Source Signal Feature Extraction Results

**Figure 12:**
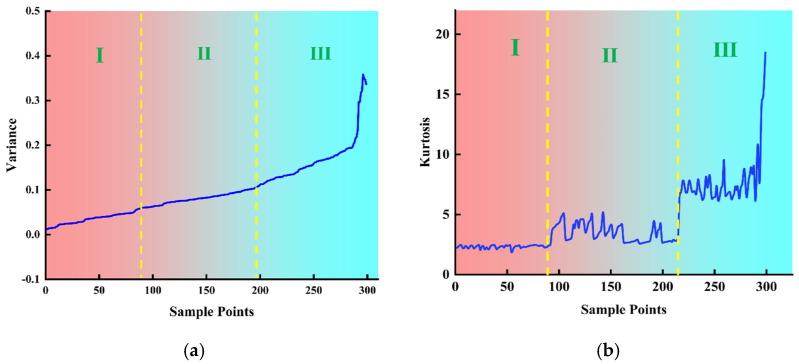

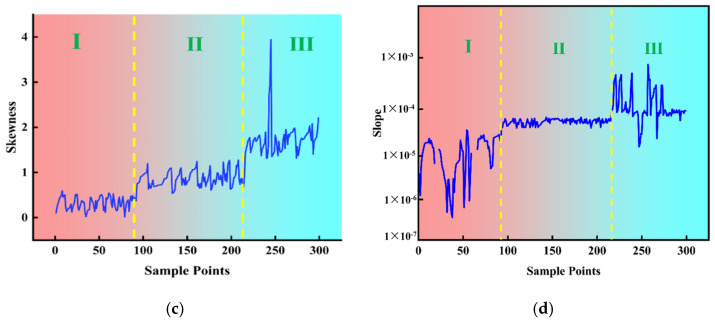
Time-frequency domain characteristics of voltage signals for different fusion states: (**a**) variance; (**b**) kurtosis; (**c**) skewness; (**d**) slope.

**Figure 13:**
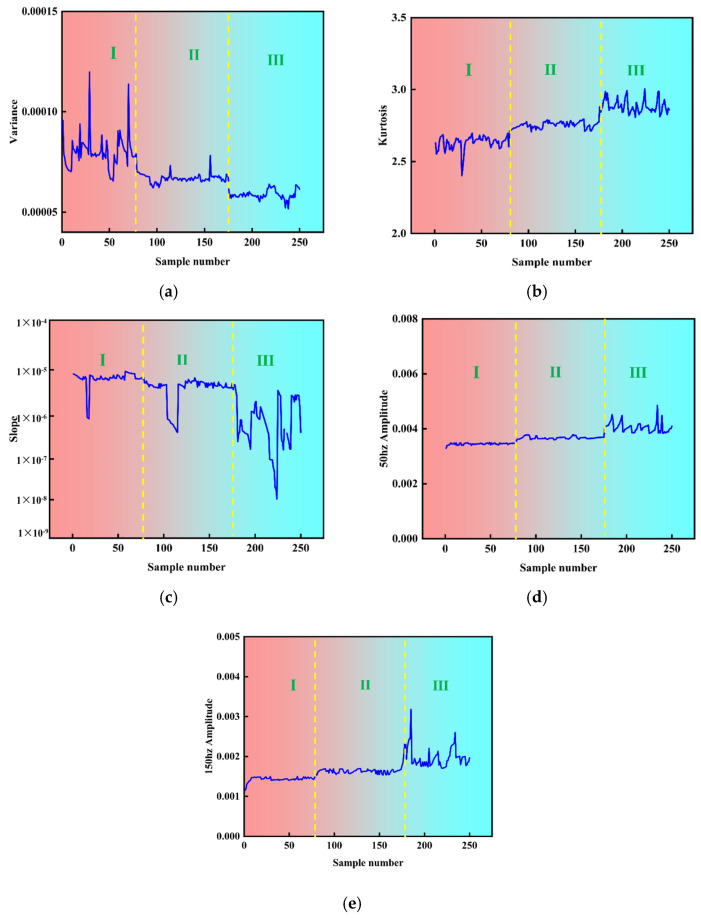
Time-frequency domain characteristics of pressure signals for different fusion states: (**a**) variance; (**b**) kurtosis; (**c**) slope; (**d**) 50 Hz amplitude; (**e**) 150 Hz amplitude.

Comprehensive GRA and PCC combination analysis (GPCC) takes into account the correlation between each feature and the ensuing outcomes, as well as the replicability across various feature types. Extraction of the features of these two signals was facilitated utilizing downscaling and dimensional extraction techniques. This approach rendered the trend of melting penetration more discernible. The parameters of the arc signal and the pressure signal were ascertained, as illustrated in Figure 12 and Figure 13. It was observed that features 1–80 remained unmelted, features 81–180 underwent complete melting, and features 181–256 were characterized by overfusing, The arc signal characteristics of fusion detection ability in descending order are variance, kurtosis, skewness, and slope, and the arc pressure signal characteristics of fusion detection ability in descending order are variance, kurtosis, slope, 50 Hz amplitude, and 150 Hz amplitude.

#### 3.3.4. Multi-Source Signal Feature Fusion Under Different Fusion States

**Figure 14:**
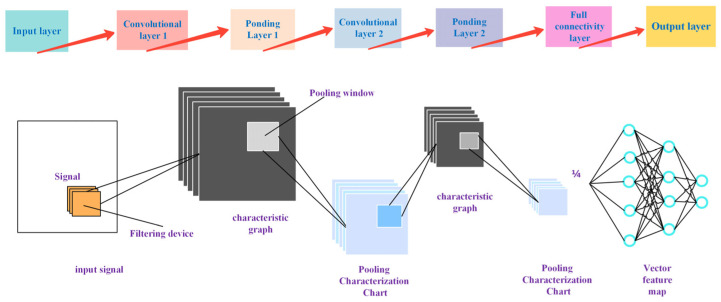
The CNN features a fusion network structure.

Convolutional neural networks (CNNs) are employed for feature fusion, as illustrated in Figure 14. This figure details the process of feature fusion analysis for the arc signal and arc pressure signal. The arc signal branch is processed by a five-layer 1D-CNN for time domain current/voltage signals. The convolution kernel is configured to have a width of 5, a step size of 2, and many channels in the order of 16, 32, 64, 128, and 256 to perform the localized discharge feature extraction. The pressure signal branch utilizes a three-layer 1D-CNN+BiLSTM hybrid structure, configured with a convolutional kernel width of 7 and 64 BiLSTM units, to capture the bidirectional time dependence of pressure fluctuations. The discrepancy between the two signals is resolved utilizing deformable convolution.

During the fusion training process, the ReLU activation function is introduced to enable the network to learn complex features. Dropout regularization is used to prevent overfitting and improve the model’s generalization ability. It is applied after the fully connected layer. The optimizer is set to Adam, which adjusts the learning rate to rapidly achieve convergence. The loss function is cross-entropy loss. It is used for classification tasks to measure the difference between the predicted probability distribution and the true distribution. The batch size and the number of training rounds directly impact the training speed and stability. Table 5 clearly shows the specific values of the model’s hyperparameters.

**Figure 15:**
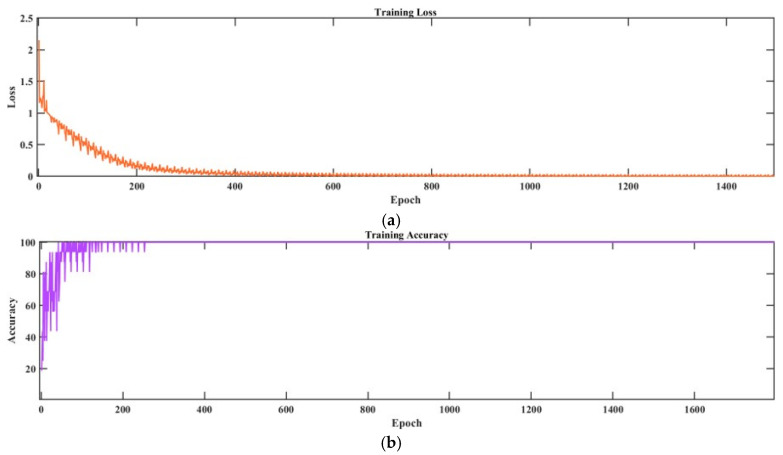
CNN feature fusion training results: (**a**) loss function; (**b**) accuracy.

As illustrated in Figure 15, the efficacy of the fused training outcomes is corroborated. The loss function curve reveals that fused modal training of the arc and pressure signals converges at a local optimum after 400 iterations (epoch) (loss ≈ 0.25), and the fused model attains stable convergence after epoch 600 (loss ≈ 0.12). These findings substantiate that the validation set exhibits an accuracy of 95.3% and that the fused feature vector is suitable for utilization as the input to the GRA-PCC-SVM fusion penetration detection model.

## 4. GPCC-CNN-SVM Fusion Penetration Recognition Modeling

### 4.1. Model Training and Validation

Given the reliability and representativeness of the data set, the experiments were conducted under identical conditions, with a signal sampling frequency of 1000 Hz and 1000 sampling points constituting one sample data. The sample data were extracted from stages I, II, and III in a stratified manner, with sample labels of 1, 2, and 3, respectively, denoting the unfused, fully fused, and overfused states. The extraction of sample data under alternative welding parameters and excitation parameters was conducted in this manner. The sample data are divided into a training set and a test set at a 7:3 ratio, which is then used for model training. The GPCC analysis yielded a total of nine features, comprising four arc features and five pressure features. These feature data were then organized into a data set comprising 252 samples, with each sample corresponding to a specific melting state label. The number of samples that remained unmelted, fully melted, and overmelted was 76, 95, and 81, respectively. The training and test sets were divided into a 7:3 ratio for model training.

To provide further evidence of the superiority of the proposed GPCC-CNN-SVM algorithm in the identification of melt, a comparison of the method with GRA-SVM, PCC-SVM, single-arc signal SVM, and single-pressure signal SVM is made, respectively. The evaluation metrics for the melt penetration data set under multi-pole magnetization include accuracy, precision, recall, and F1 score, which are the key metrics for evaluating the effectiveness of the GPCC-SVM algorithm. Accuracy is defined as the ratio of correctly predicted samples to the total number of samples. Precision indicates the proportion of samples that are positive classes out of all samples predicted to be positive classes. The F1 score, which is the reconciled mean of precision and recall, ranges from 0 to 1, with higher values indicating better prediction accuracy. Table 6 provides a comprehensive summary of the classification ability of the five melt pool fusion state models. As shown in the table, the accuracies of the data sets of GPCC-SVM, GRA-SVM, PCC-SVM, single-arc signal SVM, and single-pressure signal SVM are 98.64%, 90%, 91.33%, 88.64%, and 86%, respectively. The GPCC-SVM model demonstrates the highest accuracy and, concurrently, the models in terms of recall rate, precision, and F1 score exhibit substantial performance, suggesting that GPCC-SVM can effectively discriminate between categories. In this study, GRA is employed for the expeditious and efficacious pre-processing of the original features to efficiently eliminate features that bear minimal relevance to the fusion state, thereby reducing the feature dimension. Furthermore, based on the PCC, the elimination of redundant feature information in different fusion states is avoided, which leads to higher accuracy of GPCC-SVM than GRA-SVM and PCC-SVM. Taking the confidence level as 0.01, the five confidence intervals of accuracy are calculated as [0.9668, 1], [0.8493, 0.9507], [0.8657, 0.9609], [0.8327, 0.9401], and [0.8013, 0.9187], which indicate that the GPCC-SVM model performs well in both accuracy and estimation precision The GPCC-SVM model has the highest lower limit of the confidence interval (0.9668) and the narrowest interval width (0.0332), which indicates that its accuracy estimation is very precise and has less variability. In contrast, the other models have lower confidence interval limits and wider interval widths, indicating that their accuracy estimates are more variable and less precise than GPCC-SVM. Consequently, the developed GPCC-SVM model exhibits superior classification performance and provides a reliable foundation for the precise identification of fusion states.

**Figure 16:**
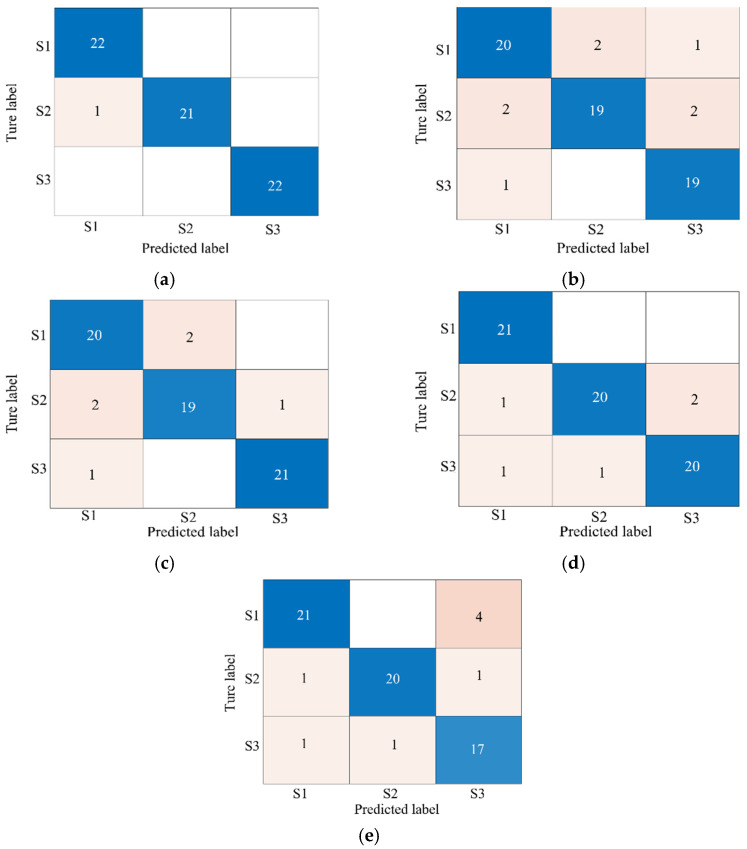
Confusion matrices: (**a**) GPCC-CNN SVM; (**b**) single-arc signal SVM; (**c**) GRA SVM; (**d**) PCA SVM; (**e**) single-pressure signal SVM.

The confusion matrices are a graphical representation of the prediction results of the models for each category. These matrices allow for the evaluation of the classification ability of different models on the test data, as shown in Figure 16. This figure corresponds to the confusion matrices of five different models: GRA-SVM, PCC-SVM, single-arc signal SVM, single-pressure signal SVM, and GPCC-CNN-SVM, respectively. In the figure, S1, S2, and S3 correspond to unfused, fully fused, and overfused, respectively. The rows of the confusion matrix represent the actual penetration states, while the columns represent the predicted penetration states. The model’s recognition performance is indicated by the proportion of diagonal elements that approach 100%. As illustrated in the figure, the models labeled b and e are single-signal recognition models, exhibiting misidentifications of eight. However, the model labeled c is a dual-signal single-optimization algorithm model, demonstrating misidentifications of six. This model exhibits higher accuracy and a higher proportion of diagonal elements compared to the single-signal recognition model, particularly for S2 and S3. The model labeled a belongs to the dual-signal and dual-feature optimization algorithm recognition model, which has the highest proportion of diagonal elements. This suggests that the GRA-SVM model performs better in recognizing these three fusion states. This enhancement is ascribed to the adaptive optimization of feature selection, which enhances the learning ability of classification recognition.

To evaluate the operational efficiency of the CNN-SVM melt-through recognition algorithms, the configuration of the industrial computer included a 2.50 GHz Intel(R) Core(TM) i5-10300H CPU with 16 GB RAM and an NVIDIA GeForce GTX 1650 GPU. The efficiency times for each type of algorithmic model are shown in Table 7. The following essay will provide a comprehensive overview of the relevant literature on the subject. The plasma arc welding process of 304 stainless steel was conducted at a sampling frequency of 1000 Hz, encompassing 1000 sample points for a duration of 1 s. The penetration duration was calculated to be 27 s. According to the tabulated data, the computation time of the five models is 115 ms or less, which fulfils the requirements of the actual working conditions and facilitates the expeditious identification of the penetration state. The GRA-SVM and PCC-SVM algorithms exhibit a one-step deficit in runtime, with the PCC-SVM algorithm demonstrating a 20-millisecond reduction in execution time. The total algorithm time of the GPCC-CNN-SVM with dual signals does not differ significantly from that of the single-arc signal SVM and single-pressure signal SVM models. However, when the accuracy of the models is taken into consideration, the GPCC-CNN-SVM with the fusion of arc and pressure signals is more capable of identifying the melt-through state in a short period of time. The CNN-SVM model has been demonstrated to adequately address the necessity for melt-through identification in the context of automatic welding.

### 4.2. Validation of Experimental Results

**Figure 17:**
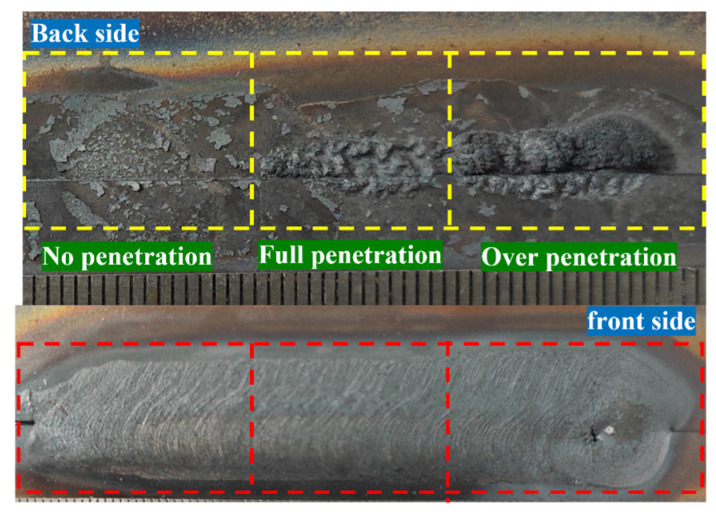
Different melt-through conditions under continuous welding conditions.

**Figure 18:**
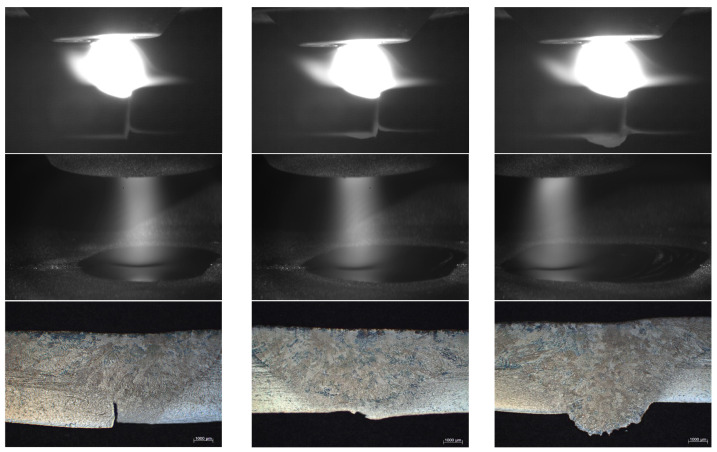
Arc pool and weld cross-section for continuous welding conditions.

**Figure 19:**
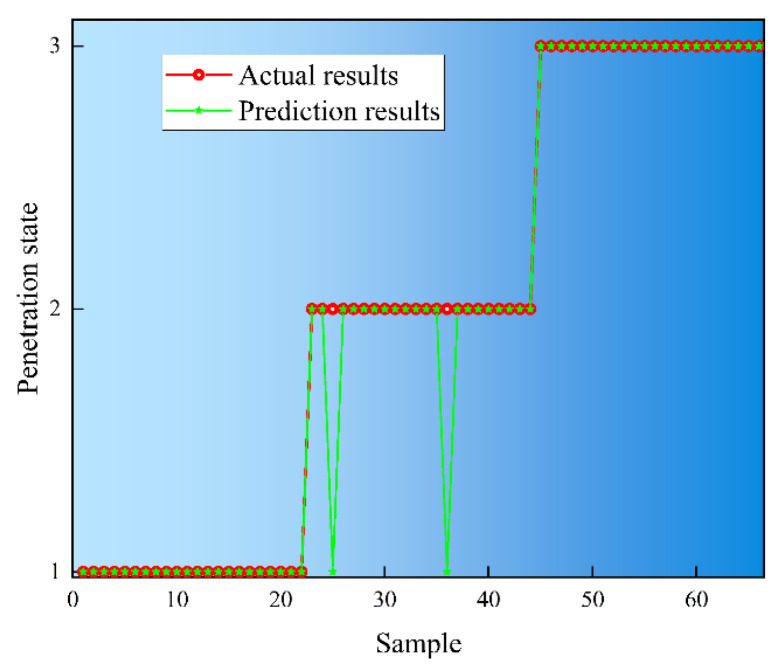
Detection results of continuous welding conditions.

Concurrently, to ascertain the efficacy of the method in detecting disparate melt penetration states during continuous welding, a series of continuous welding experiments were conducted, incorporating diverse welding processes following the welding parameters delineated in Table 1. The outcomes of these experiments are depicted in Figure 17. Figure 18 shows the arc pool and weld cross-section for continuous welding conditions. A total of 66 data sets were extracted, of which 22 were classified as unmelted, fully melted, or overmelted. The remaining 66 data sets were selected as the training set to obtain the results of fusion state detection under different fusion states, as shown in Figure 19. The regions segmented by dashed lines in the figure are, from left to right, the unmelted region, the fully melted region, and the overmelted region. In the recognition results, the unfused state corresponds to number 1, the fully fused state corresponds to number 2, and the overfused state corresponds to number 3.

As illustrated in Figure 17, the front surface of the unpenetrated weld area exhibits no signs of collapse, while the back surface of the weld displays an absence of melt zone formation. Furthermore, the detection accuracy reaches 98% in the identification of the unpenetrated specimens. In the case of the fully penetrated weld, the front face demonstrates an absence of collapse, while the back face exhibits a discontinuous melt area, characterized by discrete melt points. During the identification of the fully penetrated specimens, two sets of specimens were identified as not penetrated with 92% accuracy. This deviation can be attributed to the presence of a minor unmelted area within the fully melted region, resulting in a discrepancy between the predicted and actual fusion states. In contrast, the front side of the overmelt weld region exhibited a clear collapse, while the back side of the weld displayed a complete melt line, with a 97.3% accuracy rate for the fully melted samples. By calculating the mean detection accuracy for the three distinct penetration states, an acceptable detection accuracy of 96% was attained for the continuous weld penetration state. However, when identifying the fully melted samples, two sets of samples were identified as not melted, which is a false positive because the actual state was fully melted but incorrectly identified as not melted. This is due to the fact that there is still a small amount of unmelted area in the fully melted region, causing the actual melted state to deviate from the predicted melted state. In addition, although there were no cases of unmelted being misidentified as fully melted or overmelted, which are false negatives, the algorithm may need to be improved or more training data added to further improve the accuracy of identification.

## 5. Conclusions

In this study, a magnetic field-assisted molten pool oscillation melt penetration identification method based on GPCC-CNN-SVM is proposed for melt penetration state identification during plasma arc welding of medium-thickness 304 stainless steel sheets with a welding current of 70–100 A and a magnetic field condition of 1–3 A and 2–6 Hz working conditions. Firstly, a multi-pole magnetron is introduced to regulate the regular oscillations that occur in the arc and weld pool during the welding process. Secondly, to ensure that representative feature vectors can be extracted from the arc voltage and pressure signals, Gray relational analysis (GRA) and Pearson correlation analysis (PCC) algorithms are used to perform feature optimization on the raw signals, and the features most reflective of the melt-through state are screened out. Finally, the extracted feature vectors are fed into the SVM model for feature extraction and classification. The method improves the accuracy and robustness of detecting different melt pool states based on arc voltage signals during the welding process, and the main conclusions are as follows:

1. A multi-sensor sensing system was established to construct a weld penetration identification model. This system was based on arc pressure and arc voltage. The multi-pole magnetron-assisted distribution of temperature and pressure was analyzed, and a mapping model of the frequency change in arc and pressure signals on the weld penetration state was constructed.
2. The proposed fusion detection method for arc signal and pressure signal is a significant contribution to this study. The high-dimensional feature efficient extraction method for the fusion detection model is also proposed, and its accuracy is verified with the extracted effective features.
3. A welding fusion recognition model based on GPCC-CNN-SVM is established, 23 dimensional features, including the arc signal and pressure signal, are extracted, data dimensionality is reduced by combining GRA and PCC, and a confusion matrix and a recognition model with an accuracy rate of 98.6% are obtained by SVM training.
4. Melting experiments verified that the recognition rate of the model for the three different melt pool states of unmelted, fully melted, and overmelted was 98%, 92%, and 97.3%, respectively. The recognition rate of the molten pool state for continuous mobile welding under varying welding process conditions is 96%.

The present paper is dedicated to the development of an intelligent system for the identification of the degree of penetration in plasma arc welding, utilizing the fusion of multi-pole magnetron and arc pressure and voltage signals. Future research endeavors may encompass the training of the model with various materials, including carbon steel, aluminum alloys, and a range of process conditions. This approach aims to enhance the model’s versatility and adaptability. Additionally, there is scope to enhance signal processing and feature extraction techniques. This will lead to the development of more robust feature extraction methods, thereby reducing the impact of noise in real-world environments. The exploration of more lightweight model architectures is of particular interest in this field. Such models have the advantage of reducing computational resource requirements and improving the model’s ability to be deployed in resource-constrained environments. Through the aforementioned improvements and extensions, future work can further improve the performance and application scope of melt penetration recognition algorithms in real industrial environments. This will provide more accurate and reliable quality control means for automated welding processes.

## Figures and Tables

**Table 1:** Experimental parameters.

Welding Current I	Welding Speed *v_w_*	Welding Height *h*	Flow Rate *v_g_*
70 A	0.2 m∙min^−1^	7 mm	12 L∙min^−1^
80 A	0.2 m∙min^−1^	7 mm	12 L∙min^−1^
90 A	0.2 m∙min^−1^	7 mm	12 L∙min^−1^

**Table 2:** Time–frequency characteristics.

Feature	Formula	Feature	Formula
Mean	$\bar{x}=\sum_{i=1}^{m}x_{i}/m$	Maximum	$x_{max\;}=max\left(x_{i}\right)$
Variance	$s^{2}=\sum_{i=1}^{m}{\left(x_{i}-\bar{x}\right)}^{2}/m$	Minimum	$x_{min}=min(x_{i})$
Root Mean Square	$RMS=\sqrt{\frac{1}{n}\sum_{i=1}^{n}x_{i}^{2}}$	Peak to Peak	$x_{peak-to-peak}=x_{max}-x_{min}$
Kurtosis	$k=\frac{1}{m}\sum_{i=1}^{m}{\left(\frac{x_{i}-\bar{x}}{\sigma_{t}}\right)}^{4}$	Form Factor	$\frac{RMS}{\frac{1}{n}\sum_{i=1}^{n}\left|x_{i}\right|}$
Skewness	$S=\frac{\frac{1}{n}\sum_{i=1}^{n}{\left(x_{i}-\mu \right)}^{3}}{\sigma^{3}}$	Crest Factor	$\frac{x_{peak}}{RMS}$
Slope	$k=\frac{\Delta y}{\Delta x}$	Centroid	$C=\frac{\sum_{f}f\cdot {\left|X\left(f\right)\right|}^{2}}{\sum_{f}{\left|X\left(f\right)\right|}^{2}}$
Flatness	$\frac{\prod_{f}{\left|X\left(f\right)\right|}^{1/n}}{\frac{1}{n}\sum_{f}\left|X\left(f\right)\right|}$	Entropy	$P\left(f\right)=\frac{{\left|X\left(f\right)\right|}^{2}}{\sum_{f}{\left|X\left(f\right)\right|}^{2}}$
Mean Square Frequency	$MSF=\frac{\sum_{f}f^{2}\cdot {\left|X\left(f\right)\right|}^{2}}{\sum_{f}{\left|X\left(f\right)\right|}^{2}}$	Root Mean Square Frequency	$RMSF=\sqrt{\frac{\sum_{f}{\left(f-C\right)}^{2}\cdot {\left|X\left(f\right)\right|}^{2}}{\sum_{f}{\left|X\left(f\right)\right|}^{2}}}$

**Table 3:** Signal-to-noise ratio of the two signals.

Signal Type	Single Sensor SNR (dB)	Fused Sensor SNR (dB)	Increase Amplitude (dB)
Arc voltage	8	15	7
Arc pressure	5	12	7
Joint signal	-	18	10

**Table 4:** Comparative analysis of GRA-PCC.

Characterization	PCC	GRA
Type of Relationship	Linear Correlation	Nonlinear Correlation
Measurement Impact	None (Normalization)	Data Normalization Required
Computational Complexity	O(n)	O(n)
Physical Interpretation	Clear (Slope and Direction)	Relatively Clear
Outlier Sensitivity	Higher	Higher

**Table 5:** Hyperparameter settings.

Parameters	Values
Learning rate	0.001
Optimizer	Adam
Loss function	Cross-entropy loss
Activation function	ReLU
Epochs	1600
Batch size	32
Regularization	Dropout (rate = 0.5)

**Table 6:** Classification capabilities of the three fusion states.

Input	Model	Metrics			
		Acc (%)	Pre (%)	Rec (%)	F1 (%)
Arc+Pressure Signal	GRA-SVM	90	93.42	90	90.42
Arc+Pressure Signal	PCC-SVM	91.33	92	91.33	92
Arc Signal	Arc Signal SVM	88.64	83	88.64	83
Pressure Signal	Pressure Signal SVM	86	85.67	86	85.33
Arc+Pressure Signal	GPCC-CNN-SVM	98.64	97	97.79	97.79

**Table 7:** Runtime analysis of different recognition models.

Algorithmic Step	GPCC-CNN-SVM (ms)	GRA-SVM (ms)	PCC-SVM (ms)	Single-Arc Signal SVM (ms)	Single-Pressure Signal SVM (ms)
Gray relational analysis	25 ms	30	-	25	25
Pearson correlation analysis	40 ms	-	40	40	40
CNN-SVM training	50 ms	30	40	40	42
Total algorithm time	115	90	80	105	107

## Data Availability

Data are contained within the article.

## References

[1] Liu Z., Li X., Pan L., Gao J., Zhang K. (2023). Effects of weld penetration modes on laser welding characteristics of a novel ultra-high strength steel for aerospace application. J. Manuf. Process..

[2] Chen Z., Yu B., Wang P., Qian H. (2023). Fatigue properties evaluation of fillet weld joints in full-scale steel marine structures. Ocean Eng..

[3] Amanat N., Chaminade C., Grace J., McKenzie D.R., James N.L. (2010). Transmission laser welding of amorphous and semi-crystalline poly-ether–ether–ketone for applications in the medical device industry. Mater. Des..

[4] Kumar S.M., Kannan A.R., Pramod R., Shanmugam N.S., Dhinakaran V. (2022). Testing, characterization and numerical prediction (uni-axial tension and bend test) of Double-side TIG welded SS321 plate for pressure vessel application. Int. J. Press. Vessel..

[5] Mishra D., Pal S.K., Chakravarty D. (2021). Industry 4.0 in welding. Weld. Technol..

[6] Jiang F., Li W., Xu B., Cheng W., Zhang G., Ma X., Chen S. (2024). Variable polarity plasma arc welding: Process development and its recent developments of detecting modeling and controlling. J. Manuf. Process..

[7] Wu D., Van Nguyen A., Tashiro S., Hua X., Tanaka M. (2019). Elucidation of the weld pool convection and keyhole formation mechanism in the keyhole plasma arc welding. Int. J. Heat Mass Transf..

[8] Xu B., Chen S., Tashiro S., Jiang F., Tanaka M. (2020). Material flow analyses of high-efficiency joint process in VPPA keyhole flat welding by X-ray transmission system. J. Clean. Prod..

[9] Nguyen H.L., Van Nguyen A., Duy H.L., Nguyen T.H., Tashiro S., Tanaka M. (2021). Relationship among welding defects with convection and material flow dynamic considering principal forces in plasma arc welding. Metals.

[10] Cui Y., Shi Y., Zhu T., Cui S. (2020). Welding penetration recognition based on arc sound and electrical signals in K-TIG welding. Measurement.

[11] Wu D., Huang Y., Chen H., He Y., Chen S. (2017). VPPAW penetration monitoring based on a fusion of visual and acoustic signals using t-SNE and DBN model. Mater. Des..

[12] Wu D., Huang Y., Zhang P., Yu Z., Chen H., Chen S. (2020). Visual-acoustic penetration recognition in variable polarity plasma arc welding process using hybrid deep learning approach. IEEE Access.

[13] Gao Y., Wang J. (2022). Weld penetration identification with deep learning method based on auditory spectrum images of arc sounds. Weld. World.

[14] Gao Y., Zhao J., Wang Q., Xiao J., Zhang H. (2020). Weld bead penetration identification based on human-welder subjective assessment on welding arc sound. Measurement.

[15] Gao Y., Wang Q., Xiao J., Zhang H. (2020). Penetration state identification of lap joints in gas tungsten arc welding process based on two channel arc sounds. J. Mater. Process. Technol..

[16] Wang Q., Gao Y., Huang L., Gong Y., Xiao J. (2019). Weld bead penetration state recognition in GMAW process based on a central auditory perception model. Measurement.

[17] Zhang Y., Chen B., Tan C., Song X., Zhao H. (2023). An end-to-end framework based on acoustic emission for welding penetration prediction. J. Manuf. Process..

[18] Luo Z., Wu D., Zhang P., Ye X., Shi H., Cai X., Tian Y. (2023). Laser welding penetration monitoring based on time-frequency characterization of acoustic emission and CNN-LSTM hybrid network. Materials.

[19] Chen J., Chen J., Feng Z. (2019). Model predictive control of GTAW weld pool penetration. IEEE Robot. Autom. Lett..

[20] Li B., Shi Y., Wang Z. (2024). Penetration identification of magnetic controlled Keyhole Tungsten inert gas horizontal welding based on OCR-SVM. Weld. World.

[21] Liang R., Yu R., Luo Y., Zhang Y. (2019). Machine learning of weld joint penetration from weld pool surface using support vector regression. J. Manuf. Process..

[22] Baek D., Moon H.S., Park S.H. (2024). Optimization of weld penetration prediction based on weld pool image and deep learning approach in gas tungsten arc welding. Int. J. Adv. Manuf. Technol..

[23] Baek D., Moon H.S., Park S.H. (2024). In-process prediction of weld penetration depth using machine learning-based molten pool extraction technique in tungsten arc welding. J. Intell. Manuf..

[24] Jia C.B., Liu X.F., Zhang G.K., Zhang Y., Yu C.H., Wu C.S. (2021). Penetration/keyhole status prediction and model visualization based on deep learning algorithm in plasma arc welding. Int. J. Adv. Manuf. Technol..

[25] Yu R., Guo S., Huang Y., Wang L., Peng Y., Wang K. (2023). Measurement of weld penetration for variable-groove weldment using dual-band imaging and neural network. J. Mater. Res. Technol..

[26] Zeng Z., Yang Y., Yuan J., Qi B. (2024). Rapid inference for penetration prediction of plasma arc welding using enhanced ShuffleNetV2 and FOS-ELM. Weld. World.

[27] Li J., Zhang Y., Liu W., Li B., Yin X., Chen C. (2022). Prediction of penetration based on plasma plume and spectrum characteristics in laser welding. J. Manuf. Process..

[28] Zhang Y., Li F., Liang Z., Ying Y., Lin Q., Wei H. (2018). Correlation analysis of penetration based on keyhole and plasma plume in laser welding. J. Mater. Process. Technol..

[29] Zeng D., Wu D., Huang H., Peng B., Wei Y., Du H., Zhang P., Shi H., Lu Q., Cai X. (2025). Online identification of laser welding penetration through multi-photoelectric decomposition-reconstruction and shifted-windows-based transformer deep learning framework. Measurement.

[30] Jiao W., Wang Q., Cheng Y., Zhang Y. (2021). End-to-end prediction of weld penetration: A deep learning and transfer learning based method. J. Manuf. Process..

[31] Yu R., Cao Y., Chen H., Ye Q., Zhang Y. (2023). Deep learning based real-time and in-situ monitoring of weld penetration: Where we are and what are needed revolutionary solutions. J. Manuf. Process..

[32] Cui Y., Shi Y., Hong X. (2019). Analysis of the frequency features of arc voltage and its application to the recognition of welding penetration in K-TIG welding. J. Manuf. Process..

[33] Xu S., Yang L., Xu S. (2020). Characteristic relationship between the oscillating characteristics of plasma electrical signal and weld depth in laser deep melting welding. China Laser.

[34] Zhang S., Hu S., Wang Z. (2016). Weld penetration sensing in pulsed gas tungsten arc welding based on arc voltage. J. Mater. Process. Technol..

[35] Bai P., Wang Z., Hu S., Ma S., Liang Y. (2017). Sensing of the weld penetration at the beginning of pulsed gas metal arc welding. J. Manuf. Process..

[36] Wang F., Chen Y., Wang Q., Liu L., Alam M., Zhang X., Jiao W. (2024). Weld penetration state identification based on time series multi-source data fusion. Weld. World.

[37] She K., Li D., Yang K., Li M., Wu B., Yang L., Huang Y. (2024). Online detection of laser welding penetration depth based on multi-sensor features. Materials.

[38] Van Nguyen A., Tashiro S., Ngo M.H., Van Bui H., Tanaka M. (2020). Effect of the eddies formed inside a weld pool on welding defects during plasma keyhole arc welding. J. Manuf. Process..

[39] Zou S., Wang Z., Hu S., Wang W., Cao Y. (2020). Control of weld penetration depth using relative fluctuation coefficient as feedback. J. Intell. Manuf..

[40] Gatzen M. (2012). Influence of low-frequency magnetic fields during laser beam welding of aluminum with filler wire. Phys. Procedia.

[41] Meng X., Bachmann M., Artinov A., Rethmeier M. (2021). The influence of magnetic field orientation on metal mixing in electromagnetic stirring enhanced wire feed laser beam welding. J. Mech. Work. Technol..

[42] Zhou J., Tan H., Zhang L., Lin H., Jia J., Wei W. (2024). Numerical and experimental study of the enhanced melting and penetration capability of aluminum alloy gap bridging laser welding by alternating magnetic field. Opt. Lasers Eng..

[43] Liu F., Xu B., Song K., Tan C., Zhao H., Wang G., Chen B., Song X. (2022). Improvement of penetration ability of heat source for 316 stainless steel welds produced by alternating magnetic field assisted laser-MIG hybrid welding. J. Mater. Process. Technol..

[44] Liu L., Zhu Y., Liu R. (2023). Study of twin tungsten electrode–wire electrode indirect arc welding assisted by alternating magnetic field. J. Manuf. Process..

[45] Cervantes J., Garcia-Lamont F., Rodríguez-Mazahua L., Lopez A. (2020). A comprehensive survey on support vector machine classification: Applications challenges and trends. Neurocomputing.

[46] Jahangir H., Tayarani H., Baghali S., Ahmadian A., Elkamel A., Golkar M.A., Castilla M. (2019). A novel electricity price forecasting approach based on dimension reduction strategy and rough artificial neural networks. IEEE Trans. Ind. Inform..

[47] Qi Z., Liu Y., Song Q., Zhou N. (2021). An improved greedy reduction algorithm based on neighbourhood rough set model for sensors screening of exoskeleton. IEEE Sens. J..

[48] Muddineni V.P., Bonala A.K., Sandepudi S.R. (2020). Grey relational analysis-based objective function optimisation for predictive torque control of induction machine. IEEE Trans. Ind. Appl..

[49] Li G. (2020). A Pearson Based Feature Compressing Model for SNARE Protein Classification. IEEE Access.

